# Evaluation of Classic and Quantitative Imaging Features in the Differentiation of Benign and Atypical Lipomatous Soft Tissue Tumors Using a Standardized Multiparametric MRI Protocol: A Prospective Single-Centre Study in 45 Patients

**DOI:** 10.3390/curroncol30030252

**Published:** 2023-03-13

**Authors:** Leonhard Gruber, Christian Kremser, Bettina Zelger, Anton Schwabegger, Ena Josip, Dietmar Dammerer, Martin Thaler, Benjamin Henninger

**Affiliations:** 1 Department of Radiology, Medical University Innsbruck, Anichstraße 35, 6020 Innsbruck, Austria; 2 Department of Pathology, Medical University Innsbruck, Anichstraße 35, 6020 Innsbruck, Austria; 3 Department of Plastic, Reconstructive and Aesthetic Surgery, Medical University Innsbruck, Anichstraße 35, 6020 Innsbruck, Austria; 4 Department of Orthopaedics and Trauma Surgery, Universitätsklinikum Krems, 3500 Krems an der Donau, Austria; 5 Arthroplasty Center Munich West, Helios Klinikum, Steinerweg 5, 81241 Munich, Germany; 6 Center of Orthopaedics, Trauma Surgery and Rehabilitation Medicine, University of Greifswald, 17475 Greifswald, Germany

**Keywords:** soft tissue tumors, atypical lipomatous tumors, lipoma, MRI, imaging features, ranking

## Abstract

Background: Discrimination between benign and atypical lipomatous tumors (ALT) is important due to potential local complications and recurrence of ALT but can be difficult due to the often-similar imaging appearance. Using a standardized MRI protocol, this study aimed to rank established and quantitative MRI features by diagnostic value in the differentiation of benign and atypical lipomatous tumors and to develop a robust scoring system. Methods: Patients with clinical or sonographic suspicion of a lipomatous tumor were prospectively and consecutively enrolled from 2015 to 2019 after ethic review board approval. Histology was confirmed for all ALT and 85% of the benign cases. Twenty-one demographic and morphologic and twenty-three quantitative features were extracted from a standardized MRI protocol (T1/T2-proton-density-weighting, turbo-inversion recovery magnitude, T2* multi-echo gradient-echo imaging, qDIXON-Vibe fat-quantification, T1 relaxometry, T1 mapping, diffusion-weighted and post-contrast sequences). A ranking of these features was generated through a Bayes network analysis with gain-ratio feature evaluation. Results: Forty-five patients were included in the analysis (mean age, 61.2 ± 14.2 years, 27 women [60.0%]). The highest-ranked ALT predictors were septation thickness (gain ratio merit [GRM] 0.623 ± 0.025, *p* = 0.0055), intra- and peritumoral STIR signal discrepancy (GRM 0.458 ± 0.046, *p* < 0.0001), orthogonal diameter (GRM 0.554 ± 0.188, *p* = 0.0013), contrast enhancement (GRM 0.235 ± 0.015, *p* = 0.0010) and maximum diameter (GRM 0.221 ± 0.075, *p* = 0.0009). The quantitative features did not provide a significant discriminatory value. The highest-ranked predictors were used to generate a five-tiered score for the identification of ALTs (correct classification rate 95.7% at a cut-off of three positive items, sensitivity 100.0%, specificity 94.9%, likelihood ratio 19.5). Conclusions: Several single MRI features have a substantial diagnostic value in the identification of ALT, yet a multiparametric approach by a simple combination algorithm may support radiologists in the identification of lipomatous tumors in need for further histological assessment.

## 1. Introduction

Lipomatous tumors (LT) are the most common soft tissue neoplasms [1,2,3] and among the most common tumors encountered in clinical practice [4,5]. Their lipomatous nature can readily be established in most cases by imaging [4]. Still, LT encompass a wide imaging spectrum ranging from overtly dedifferentiated lesions without imaging features betraying their lipomatous origin such as dedifferentiated liposarcoma (DDLS) to highly differentiated masses almost indistinguishable from the surrounding fat tissue such as benign lipomas (BL) or atypical lipomatous tumors (ALT) [1,4]. Especially in the latter group, distinguishing between benign lipomas and ALT can be challenging for clinicians, radiologists and pathologists [6]. Nonetheless, this differentiation is essential, as intermediate lesions can show local recurrence [3,7,8,9], requiring a reliable initial diagnosis and complete resection. Magnetic resonance imaging (MRI) is the modality of choice for the evaluation of lipomatous lesions [10], as high resolution, tissue contrast, and functional tissue properties provide the reader with a wealth of information. Still, low inter-reader agreement has been reported [8], as BL and ALT can share imaging features such as the non-adipose content [10]. 

Several publications have discussed imaging features to reliably differentiate between BL and ALT, focusing on localization, morphology and MRI signal properties [8,11,12], highlighting the diagnostic value of several predictors such as lesion contour, size and contrast enhancement [10,12] and special sequences such as short-tau inversion recovery (STIR) [13]. Several quantitative MRI sequences such as T2* and relaxometry sequences have seen application for the evaluation of soft tissue tumors over the last years [14,15,16,17]. Still, the low sensitivity and specificity of the MRI evaluations in ambiguous cases [18] often necessitate a diagnosis by histopathological examination [8], which has made great diagnostic strides on the basis of molecular analyses such as MDM2/CDK4 amplification analysis [3,19]. Prior studies [14,15,16,17] on imaging features have relied on conventional statistics reporting of predictor properties such as sensitivity, specificity and odds ratios (OR), often not reflecting the multitude of parameters simultaneously weighed by musculoskeletal imaging specialists during reading [12]. Bayesian approaches allow modeling predictor interactions and yield weighed rankings, decision trees or complex conditional networks [20]. 

The purpose of this study was to evaluate a standardized MRI protocol for lipomatous soft tissue tumor assessment including established morphological and quantitative sequences (T1 mapping and tissue relaxation times), evaluate single predictor performance and, on the basis of a Bayes network analysis, develop a generalizable and easy-to-follow score to differentiate benign lipomas from ALT.

## 2. Materials and Methods

### 2.1. Approval by the Ethical Review Board

Study approval was granted by the Ethical Review Board of the Medical University Innsbruck (proposal AN2015-0289 356/4.9).

### 2.2. Patient Recruitment

The inclusion criteria were as follows: (1) referral for an MRI due to an unknown soft tissue mass with suspected lipomatous differentiation upon clinical or ultrasonographic inspection, (2) confirmed soft tissue tumor of lipomatous differentiation according to the WHO guidelines [21] or lipomatous tumor without imaging changes and tumor board review after at least twelve months, (3) patient age 18 years or above, (4) read and signed formal consent. The exclusion criteria were: (1) MRI or contrast agent contraindications, (2) age below 18 years, (3) pregnancy. The patients were included in a consecutive fashion. All participants gave their informed written consent after a detailed description of study protocol deviations from routine imaging, including additional time within the scanner. No adverse events of any grade occurred.

### 2.3. Histopathological Analysis

Histopathological analysis was performed by B.Z. (30 years of experience) following international guidelines. All tissue samples were fixed in a buffered 4% formaldehyde solution and embedded in paraffin (FFPE) for routine clinical pathological diagnostics according to diagnostic standards. Preparation of hematoxylin-and-eosin-stained slides as the basic gold standard in pathological diagnosis was performed in each case. Automatized immunohistochemistry with the primary antibodies anti-MDM2 (Abcam EPR1450 2), -CDK4 (Abcam EPR4513-32-7) and -p16 (clone DCS-50, antikoerper-online.de) was conducted on a Ventana Benchmark Ultra. Fluorescence in situ hybridization was performed on 4 mm thick FFPE tumor slides to evaluate MDM2 amplification (Vysis MDM2/CEP 12 FISH Probe Kit).

### 2.4. MRI Protocol

MR imaging was performed on a 1.5T whole body MR scanner (MAGNETOM Avanto^fit^, Siemens Healthcare, Erlangen, Germany). The patients were scanned in supine position. The receive coils were selected according to the respective body region examined and always consisted of a matrix of phased-array coil elements. The examination protocol consisted of the following sequences: (1) turbo inversion recovery magnitude (TIRM), (2) T1w turbo spin echo sequence without fat suppression before and after contrast agent application, (3) dual-echo (proton density [PD]/T2w) turbo spin echo sequence for T2 estimation, (4) 3D multi-gradient echo sequence (qDixon-Vibe) with automatic calculation of proton density fat fraction maps based on a multi-fat-peak model after manual drawing of the single-slice region of interest (ROI) [22], (5) readout-segmented echo-planar diffusion-weighted sequence (RESOLVE) with five different b-values (0, 50, 200, 400, 800 s/mm²) and four diffusion directions (4-scan trace), (6) T1 mapping, (7) 2D multi-echo gradient echo (meGRE) [23], and (8) T1 VIBE Dixon before and after intravenous gadolinium administration. The detailed sequence parameters are shown in Table 1.

### 2.5. Patient Characteristics and Imaging Features

The evaluation of the MR images was performed by 2 experienced radiologists in consensus (LG: six-year experience in musculoskeletal imaging, BH: eleven-year experience in musculoskeletal imaging) in a joint meeting, blinded to all available histological data. All observations were determined in consensus. Primary measurements were performed in our institution’s picture archiving and communication system (PACS) application Impax EE (Agfa, Mortsel, Belgium).

The demographic and lesion parameters included in the analysis are presented in Supplementary Table S1. 

### 2.6. Gray Value Analysis of T1, T2, T2*, R2* and ADC Maps

A histogram analysis including mean, standard deviation, minimum and maximum values was performed for T1, T2, T2*, R2* and ADC maps. The fat fractions were calculated from qDIXON-Vibe sequences.

### 2.7. Statistics

All data were stored in Microsoft Excel 16.16.21 (Microsoft; Redmond, DC, USA). The statistical software used were GraphPad Prism 9.0.0 (GraphPad Software LLC; La Jolla, CA, USA) and WEKA 3.8.4 (University of Waikato; Waikato, New Zealand) [24]. 

Overall, the tumors were separated into a benign (B) and an atypical lipomatous tumor (ALT) group. 

Depending on group count and distribution, continuous data of both groups were compared via an unpaired *t*-test or the Mann–Whitney-*U* test (in case of non-Gaussian distribution); in case of multiple comparisons, an ordinary one-way ANOVA with a Holm–Sidak correction or the Kruskal–Wallis test with Dunn’s post-test (in the case of a non-Gaussian distribution) was performed. Normality was assessed by the Kolmogorov–Smirnov test. If possible, a logarithmic transformation was performed to achieve normality for variables with a non-Gaussian distribution. The categorical variables were compared via the Fisher’s exact test. 

Invalid or missing data were discharged from further analysis, and no imputation techniques for missing data were applied. Statistical significance was considered when the *p*-value was <0.05.

To assess the correlation between imaging features and lesion differentiation (B vs. ALT), a Bayes network analysis with a 30-fold cross-validation was used. The model results include correct classification rate (CCR), mean absolute error (MAE) and weighted average for the true positive rate (TP_avg_), false positive rate (FP_avg_), precision (P_avg_) and receiver-operator characteristics (ROC) area under the curve (AUC). To generate a predictor ranking, the gain ratio feature evaluation with a ranker algorithm was used within WEKA. The predictor results included gain ratio, ALT rates and odds ratios (OR) including 95% confidence intervals (CI) calculated from contingency tables. In the case of continuous or ordinal variables, the ideal cut-off was determined using receiver-operating characteristics (ROC) curves and Youden’s J to convert them into binary variables. 

Based on the above analysis, a six-tiered score (ranging from 0 to 5 points) based on the five highest-ranked predictors was developed to identify atypical lipomatous tumors. The cut-off values for the continuous variables were determined based on ROC analysis and Youden’s index. The results are presented as box-plot, ROC-AUC and *p*-value.

## 3. Results

### 3.1. Lesion Characteristics

Overall, 45 patients could be prospectively included from June 2015 to August 2019. Three participants had to be excluded after histopathological examination revealed a non-lipomatous tumor. Please refer to Table 2 for demographic information. No difference in regard to age (61.7 ± 14.1 vs. 57.9 ± 17.2 years, *p* = 0.546) or female gender (59.0% vs. 66.7%, *p* > 0.999) was found between B and ALT patients.

On average, the tumors had a length of 104.6 ± 54 mm, a width of 64.7 ± 29.5 and a short-axis diameter of 40.1 ± 22.1 mm. The most frequent lesion locations were the shoulder (20.9%), chest wall (18.6%) and gluteal/hip region (9.3%), followed by the neck (7.0%); 37.8% of the tumors were located subcutaneously.

In total, 86.7% of the cases were histologically confirmed, with 32 lesions (71.1%) excised in toto, one open biopsy (2.2%) and six core-needle biopsies (13.3%). All six tumors diagnosed as ALT were resected in toto. Thirty-nine tumors were classified as benign, 33 after histopathologic confirmation (18.2% CNB [*n* = 6], 3.0% open biopsy [*n* = 1], 78.8% full resection [*n* = 26]). For six lesions (13.3%), the classification as lipoma was based on clinical history, unchanged follow-up MRI at least twelve months after the initial diagnosis and interdisciplinary tumor board agreement. 

### 3.2. Lesion Dimensions

**Figure 1:**
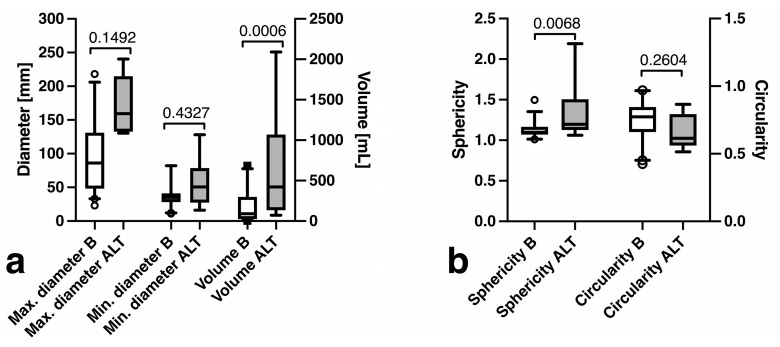
Box plots comparisons of (**a**) maximum/minimum diameters and volume and (**b**) sphericity and circularity of benign (B) tumors and atypical lipomatous tumors (ALT).

There was no significant difference in regard to minimum (37.6 ± 18.6 vs. 56.3 ± 39.3 mm, *p* = 0.149) or maximum (94.3 ± 49.2 vs. 171.2 ± 43.4 mm, *p* = 0.007) tumor diameters, yet the benign lesions (166.9 ± 189.3 mL) were significantly smaller in volume than ALT (648.7 ± 757.8 mL, *p* = 0.0006) (Figure 1a). Furthermore, sphericity was significantly lower in benign tumors (1.14 ± 0.11 vs. 1.35 ± 0.42, *p* = 0.433), while circularity did not differ significantly (0.75 ± 0.14 vs. 0.66 ± 0.13, *p* = 0.260) (Figure 1b).

### 3.3. Morphology and Descriptive Signal Characteristics

**Figure 2:**
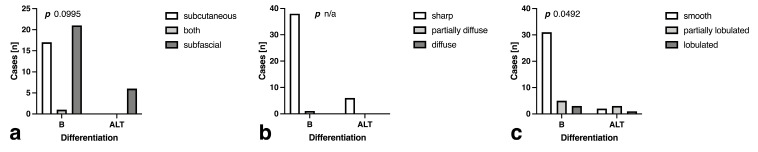
Bar plots demonstrating the distribution of (**a**) localization, (**b**) border definition and (**c**) border contour for benign (B) tumors and atypical lipomatous tumors (ALT).

Localization was no significant indicator of lesion differentiation, as subcutaneous and subfascial localization was as frequent in benign lesions, even though all ALTs were encountered subfascially (Figure 2a, *p* = 0.100). Fifty percent of ALT were encountered in the thigh or calf, while only a minority of benign tumors was found there (7.7%) (*p* = 0.037). In contrast to border definition, which was sharp in almost all cases (Figure 2b, *p* = n/a), significant lobulation correlated with a higher likelihood of ALT presence (Figure 2c, *p* = 0.049).

**Figure 3:**
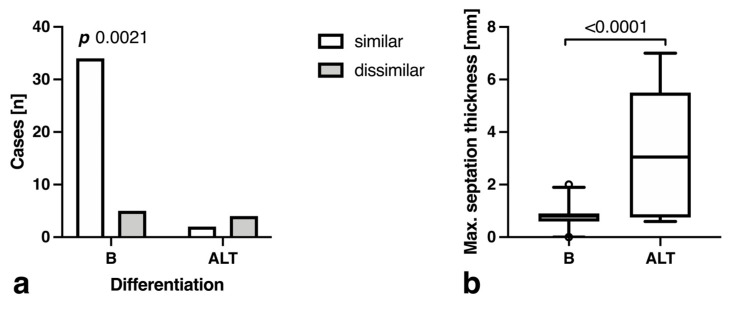
Bar plots demonstrating (**a**) septation morphology compared to surrounding fatty tissue and (**b**) maximum septation thickness in benign (B) tumors and atypical lipomatous tumors (ALT). Outliers are depicted as circles.

Cases with atypical septation morphology (thickening, nodularity) were more likely to be ALT (*p* = 0.002, Figure 3a), and this was mirrored by a significantly higher maximum septation thickness in ALT (3.25 ± 2.65 mm vs. 0.80 ± 0.45 mm, *p* < 0.0001) (Figure 3b).

**Figure 4:**
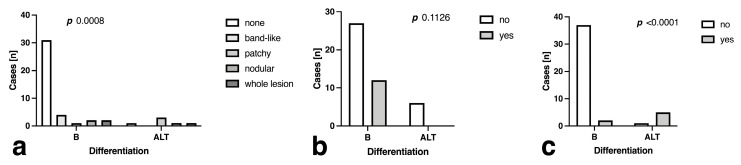
Comparison of (**a**) intralesional short-tau inversion recovery (STIR) signal distribution, (**b**) presence of surrounding STIR signal and (**c**) presence of intratumoral signal without surrounding fluid in benign (B) tumors and atypical lipomatous tumors (ALT).

Intralesional fluid accumulation patterns as assessed by STIR imaging did not differ significantly between benign tumors and ALT (*p* = 0.099, Figure 4a), even though the absence of intralesional STIR hyperintensity was significantly more likely in benign tumors (*p* = 0.009). The perilesional STIR signal was not a significant predictor for ALT differentiation (*p* = 0.1126, Figure 4b), yet the presence of intralesional STIR hyperintensity without perilesional STIR signal was highly indicative of ALT presence (*p* < 0.0001, Figure 4c).

**Figure 5:**
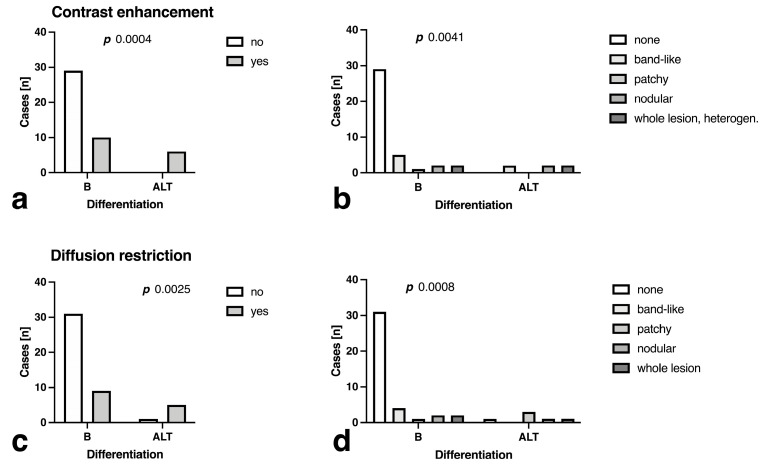
Comparison of (**a**) presence of contrast-enhancement, (**b**) contrast-enhancement patterns and (**c**) ADC alterations, defined as areas of discernible low ADC signal, (**d**) ADC pattern distribution in benign (B) tumors and atypical) lipomatous tumors (ALT.

All ALT lesions showed contrast enhancement, while only a fourth of the B lesions did so (*p* = 0.0005, Figure 5a). The contrast enhancement patterns demonstrated a significant association with differentiation, with the lack of contrast enhancement indicating benignity, while especially nodular and whole lesion enhancement favored ALT differentiation (*p* = 0.005, Figure 5b). 

Furthermore, most ALT exhibited focal, predominantly patchy, ADC alterations, whereas the majority of benign lesions showed no areas of discernible ADC signal (Figure 5c,d). 

### 3.4. Fat Fraction, T1, T2, T2*, R2* and ADC Maps Analysis

**Figure 6:**
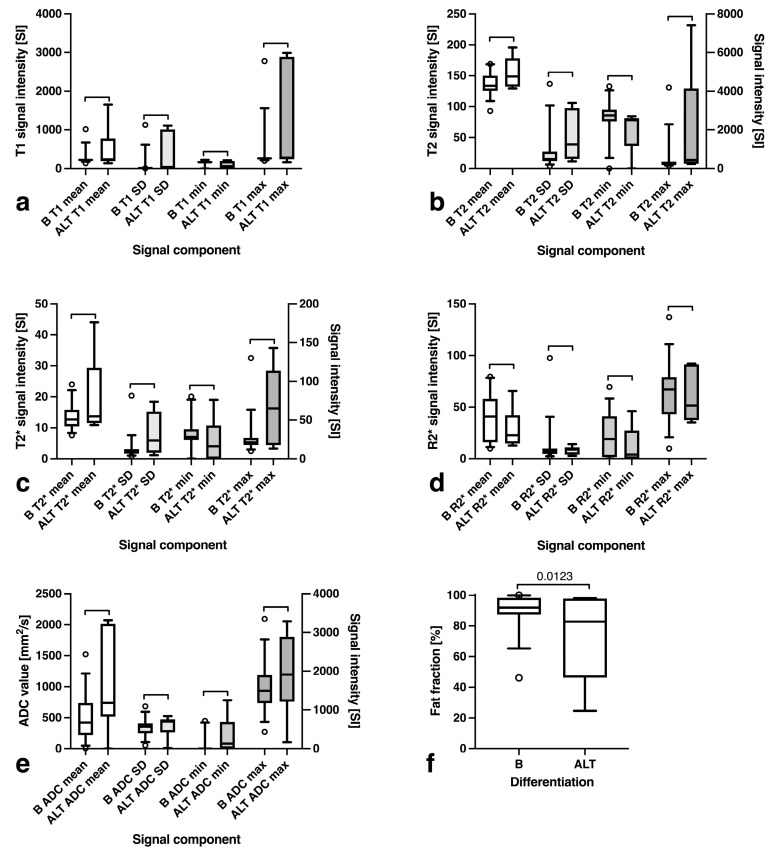
(**a**) Comparison of T1w, (**b**) T2w, (**c**) T2*, (**d**) R2* and (**e**) ADC signal intensity values as well as (**f**) fat fractions for benign (B) tumors and atypical (ALT) lipomatous tumors (ALT). Outliers are depicted as circles.

No significant difference in average T1w SI (*p* = 0.482, Figure 6a) or T2w SI (*p* = 0.906, Figure 6b) was encountered, even though the maximum T2 values were significantly higher for ALT (6.6 ± 1.4 vs. 5.7 ± 0.7 SI, *p* = 0.015) with an overall higher standard deviation (3.6 ± 0.7 vs. 2.9 ± 0.7 SI, *p* = 0.024). The same was true for T2* imaging, with a significantly higher standard deviation (1.7 ± 1.1 vs. 0.9 ± 0.6 SI, *p* = 0.005) and significantly higher maxima (3.9 ± 1.0 vs. 3.1 ± 0.5 SI, *p* = 0.005) (Figure 6c), while relaxometry showed no differing tumor properties (Figure 6d). The average ADC values were significantly higher for ALT (1039.0 ± 819.9 vs. 487.1 ± 356.3 mm^2^/s, *p* = 0.0126), owing to fat suppression (Figure 6e). There was a significant difference in fat fraction between benign tumors (90.2 ± 10.6%) and ALT (73.3 ± 30.3, *p* = 0.012) (Figure 6f).

### 3.5. Predictor Ranking

A Bayes network predictor analysis with a 30-fold cross validation showed CCR of 91.1%, MAE of 0.1037, TP_avg_ of 91.1%, FP_avg_ of 15.5%, P_avg_ of 92.7% and ROC AUC of 0.893. The predictor ranking according to gain ratio merit can be found in Table 3.

**Figure 7:**
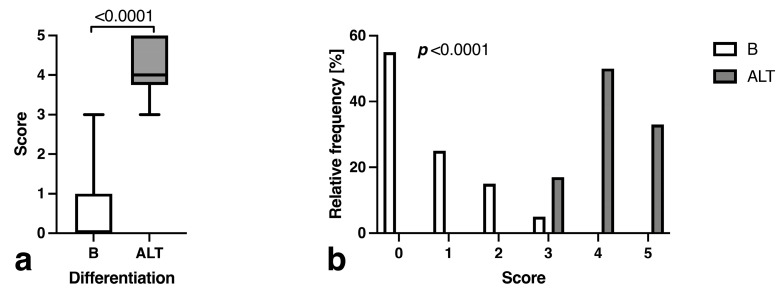
Comparison of (**a**) average score based on tumor volume, sphericity, maximum septation thickness, intra-tumoral without peritumoral STIR signal, presence of contrast enhancement and ADC signal alterations and (**b**) distribution of cases with benign or atypical differentiation by their score.

Based on this predictor ranking, a score based on maximum (cut-off 125.5 mm) and orthogonal second-largest diameter (cut-off 39.5 mm), contrast enhancement, maximum septation thickness (cut-off 1.3 mm) and intratumoral fluid accumulation without surrounding fluid was developed. The benign lesions showed a significantly lower average score of 1.4 (95% CI 1.1 to 1.7) compared to the ALT score of 4.3 (95% CI 3.5 to 5.2; *p* < 0.0001) (Figure 7a) for ROC AUC of 0.99 (*p* = 0.0001) and opposite distributions of B and ALT lesions along increasing score numbers (*p* < 0.0001; Figure 7b). The correct classification rate at a cut-off of 3 or greater was 95.7% (sensitivity, 100.0 [61.0 to 100.0%], specificity, 94.9 [83.1 to 99.1%], likelihood ratio, 19.5). 

## 4. Discussion

In this prospective study, we could demonstrate the diagnostic value of several predictors for the differentiation of benign and atypical lipomatous tumors. As described before, the advantages of an imaging-guided approach are low complication rates, avoidance of a potentially painful or debilitating procedures and cost-effectiveness in the diagnosis of soft tissue tumors [17]. Following a multivariate analysis approach, high tumor volume, atypical septation, septation thickness greater 1.3 mm, discrepancy of intratumoral STIR without surrounding fluid signal, contrast enhancement and ADC alterations were all indicative of ALT. These findings could mostly be confirmed via a Bayes network analysis. A simple algorithm based on the top five predictors to identify ALT demonstrated significant diagnostic accuracy. 

Age and gender did not differ between tumor subtypes. There were several predictors aiding in the differentiation of benign tumors from atypical lipomatous tumors, as described before [4,8,18], some reaching high positive and negative predictive values. In general, even the top 10-ranked predictors offered moderate negative predictive values, and only intralesional/surrounding STIR signal mismatch reached a sensitivity above 70%.

In general, ALT tend to be larger concerning their maximum and minimum diameters, and even though not reaching statistical significance in a multivariate analysis, the maximum and orthogonal second-largest tumor diameters ranked among the top five predictors in the differentiation when following a Bayes approach. We arrived at a cut-off of 125 mm for tumor length, similar to previous findings, that placed the cut-off at 130 mm [25]. Conversely, while the ALT volumes were significantly greater than those of benign lesions, this predictor ranked comparatively low. Similar to a previous report on soft tissue tumors [26], tumor sphericity was significantly higher for ALTs, yet ranked not very high overall. Notably, when compared to other previous reports [8,13], the tumors in our cohort were moderately sized and more often encountered superficially. Tumors of the calf or thigh were more likely to constitute ALT, in line with the literature [12].

A lower fat fraction was also correlated with ALT differentiation, in line with a higher cellularity and higher fibrous content in those subtypes [14]. Fat necrosis may mimic such alterations, though [27]. Still, fat fraction assessment ranked only moderately high after Bayes analysis.

**Figure 8:**
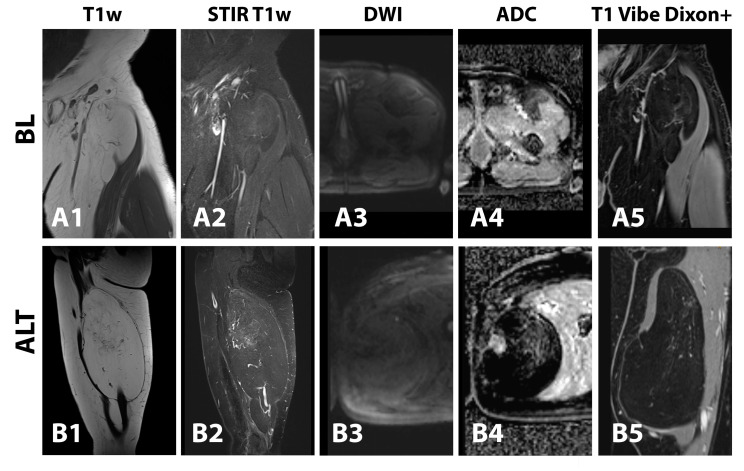
Examples of a benign (BL, (**upper row**)) and an atypical lipomatous tumor (ALT, (**lower row**)) in T1-weighted (**1st column**), short-tau inversion recovery T1-weighted (**2nd column**), diffusion-weighted (**3rd column**) images, ADC maps (**4th column**) and T1 VIBE Dixon images after intravenous contrast administration (**5th column**). Both tumors are predominantly composed of fat, yet the larger ALT shows intratumoral areas of patchy T1w signal decrease, STIR signal increase, atypical septation and patchy contrast enhancement.

In contrast to recently published findings [8], contrast enhancement was a relevant predictor of ALT differentiation even after a Bayes analysis, more in line with other studies on the topic [12,14,25]. The presence of contrast uptake alone was strongly suggestive, as were nodular or heterogeneous whole-lesion enhancement patterns, underlining the role of VEGF-based neoangiogenesis in ALTs [28]. An intralesional STIR signal corresponding to tissue water content did not identify ALTs, neither did the presence of surrounding fluid. A combination of both elements—intralesional *without* a surrounding STIR signal—though, demonstrated high sensitivity and specificity for ALT, similar to a previous study by Donners et al. [13]. Benign lipomas may mimic well-differentiated liposarcoma in certain cases, with signal increase after fat suppression, especially in case of traumatization or fat necrosis [12,29]. Our data suggest that a combinatory assessment of the intra- and peritumoral space could reduce the number of false positives in that regard (Figure 8).

Another ALT hallmark was a visually perceived thickening of septation morphology, which ranked high in our analysis, as reported before [18]. Septal thickening ranked the highest in the Bayes network analysis, even at a cut-off of 1.3 mm, a value slightly lower than reported before [8]. 

Due to fat saturation, artificially low ADC values were encountered in benign tumors. ALTs showed higher signal values, most likely resulting from tissue not affected by STIR-based fat suppression to that degree. Still, a pattern-based approach—considering the aforementioned peculiarities—proved to be highly specific and substantially sensitive for ALTs. 

As inter-reader discordance in the diagnosis of lipomatous tumors can be significant even for experienced readers regardless of the plethora of available demographic and imaging features [8], statistical methods have long been used to identify the relevant predictors in lipomatous tumors [11]. Bayes-based statistical models calculate complex probabilistic interdependencies and can reach the performance of experienced readers after sufficient training [30]. We used a Bayes network analysis to reduce the list of all assessed predictors to a small number, while retaining significant diagnostic accuracy. By combining the five predictors that ranked the highest, a simple algorithm based on septal thickening, STIR signal distribution, contrast enhancement and tumor dimensions was developed. This algorithm showed a high diagnostic accuracy and should aid radiologists and clinicians in the MRI assessment of newly diagnosed lipomatous tumors.

### Limitations

There are some limitations to report. Due to consensus reading, no interobserver correlation was performed. The proposed algorithm cannot further discriminate between histological subtypes of atypical lipomatous tumors, which still requires specialists in musculoskeletal radiology. Not all patients underwent full tumor resection, opening the possibility to core-needle biopsy or open resection sampling errors. The classification as benign was based on a follow-up of at least twelve months without histological confirmation in some cases. The benign tumors outweighed the ALT significantly compared to other studies, with a more even differentiation distribution, most likely due to consecutive study inclusion in our case—even though, in clinical routine, benign lipomas are also encountered more commonly. Reading was performed in a consensus fashion, so no data on interreader agreement can be provided. Given that all features are routinely used or derive from routinely used MRI parameters, feature robustness can be assumed. A degree of overfitting may occur in the Bayes network analysis, and our results should be confirmed in a larger dataset.

## 5. Conclusions

An intralesional/surrounding STIR signal mismatch appears to be the most robust single predictor for the presence of an ALT. A generalized multiparametric approach is able to yield substantial diagnostic accuracy in the differentiation of benign from atypical lipomatous tumors based on a simple five-tiered algorithm including septal thickening, contrast enhancement, STIR signal distribution and tumor dimensions.

## Figures and Tables

**Table 1:** MRI sequence parameters’ details.

	STIR T1w TSE	T1w TSE	PDw/T2w TSE	3D qDIXON-VIBE ^+^	RESOLVE-DWI	T1 Mapping	2D meGRE	T1 Vibe Dixon −/+
Orientation	sagittal	sagittal	axial	axial	axial	3.9	200	axial
TR (ms)	3350	644	5590	15.8	4370	1.8	1.01–19.05	6.4
TE (ms)	23	11	32/85	2.38/4.76/7.14/ 9.52/11.9/14.28	46	4–3700	-	2.39
TI (ms)	160	-	-	-	-	none	CHESS	-
Fat saturation	STIR	none	none	none	CHESS	1	1	none
Echo train length	8	6	6	6	18	4	20	2
Flip angle (°)	90	90	90	4	90	250 × 250	250 × 250	we10
FOV (mm × mm) ^#^	300 × 300	300 × 300	170 × 170	170 × 143	170 × 170	128 × 64	128 × 128	250 × 250
Acquisition matrix	512 × 410	640 × 512	384 × 269	128 × 101	88 × 62	128 × 128	128 × 128	256 × 192
Reconstructed matrix	512 × 410	640 × 512	384 × 269	256 × 202	88 × 88	5.0	10.0	256 × 256
Slice thickness (mm)	4.0	4.0	5.0	3.5	5.0	2.0 × 2.0 × 5.0	2.0 × 2.0 × 10.0	1
Voxel size (mm × mm × mm)	0.6 × 0.6 × 4.0	0.5 × 0.5 × 4.0	0.4 × 0.4 × 5.0	0.7 × 0.7 × 3.5	1.9 × 1.9 × 5.0	1	1	1.0 × 1.0 × 1.0
NEX	1	1	1	1	1	1	1	
Parallel imaging mode	GRAPPA ^$^	GRAPPA ^$^	GRAPPA ^$^	CAIPIRINHA *	GRAPPA	none	GRAPPA	GRAPPA
Accel. factor	3	3	2	4	2	-	2	3

^#^—FOV was adjusted according to the respective body region, ^+^—volumetric interpolated breath-hold, ^$^—GeneRalized Autocalibrating Partial Parallel Acquisition, *—controlled aliasing in parallel imaging results in higher acceleration.

**Table 2:** Demographic information.

	Overall	Benign (B)	Atypical Lipomatous Tumors (ALT)	*p*-Value
Participants [*n*]	45	39	6	n/a
Age [years] (mean ± SD)	61.2 ± 14.2	61.7 ± 14.1	57.9 ± 17.2	0.546 *
Female sex [*n*,%]	27 (60.0%)	23 (59.0%)	4 (66.7%)	>0.999 ^§^

* unpaired *t*-test, ^§^ Fisher’s exact test.

**Table 3:** Gain-ratio merit predictor ranking for ALT differentiation in lipomatous tumors (absolute values in parentheses).

Predictor	Avg. Rank	Gain Ratio Merit	Lipoma	ALT	Sensitivity (%)	Specificity (%)	PPV ^$^	NPV ^$^	*p*-Value ^$^
Septation thickness (cut-off 1.3 mm)	1.6 ± 0.49	0.623 ± 0.025	0.8 ± 0.4 mm	3.3 ± 2.6 mm	90.0 [77.0 to 96.0]	66.7 [30.0 to 94.1]	94.7 [82.7 to 99.1]	50.0 [21.5 to 78.5]	0.0055
STIR discrepancy (intralesional vs. surrounding)	3.0 ± 0.37	0.458 ± 0.046	5.1% (2)	83.3% (5)	95.0 [83.5 to 99.1]	83.3 [43.7 to 99.2]	97.4 [86.8 to 99.9]	71.4 [35.9 to 94.9]	<0.0001
Size (Y) (cut-off 39.5 mm)	3.0 ± 5.02	0.554 ± 0.188	37.6 ± 18.1 mm	56.3 ± 39.3 mm	95.0 [83.5 to 99.1]	66.7 [30.0 to 94.1]	95.0 [83.5 to 99.1]	66.7 [30.0 to 94.1]	0.0013
Contrast enhancement [yes/no]	4.8 ± 0.72	0.235 ± 0.015	25.6% (10)	100.0% (6)	74.4 [58.9 to 85.4]	100.0 [61.0 to 100.0]	100.0 [88.3 to 100.0]	37.5 [18.5 to 61.4]	0.0010
Size (X) (cut-off 125.5 mm)	5.5 ± 3.53	0.221 ± 0.075	94.3 ± 49.2 mm	171.2 ± 43.4 mm	75.0 [59.8 to 85.8]	100.0 [61.0 to 100.0]	100.0 [88.7 to 100.0]	37.5 [18.5 to 61.4]	0.0009
Differing septation morphology	6.4 ± 0.99	0.168 ± 0.026	12.8% (5)	66.7% (4)	87.5 [73.9 to 94.5]	66.7 [30.0 to 94.1]	94.6 [82.3 to 99.0]	44.4 [18.9 to 73.3]	0.0095
Contrast enhancement pattern (patchy, nodular, whole lesion)	6.9 ± 0.85	0.158 ± 0.012	12.8% (5)	66.7% (4)	72.5 [57.2 to 83.9]	100.0 [61.0 to 100.0]	100.0 [88.3 to 100.0]	35.3 [17.3 to 58.7]	0.0013
ADC pattern	7.6 ± 0.91	0.155 ± 0.019	23.1% (9)	83.3% (5)	75.0 [59.8 to 85.8]	100.0 [61.0 to 100.0]	100.0 [88.7 to 100.0]	37.5 [18.5 to 61.4]	0.0009
Subfascial localization	9.1 ± 0.6	0.099 ± 0.006	53.9% (21)	100.0% (6)	75.0 [59.8 to 85.8]	100.0 [61.0 to 100.0]	100.0 [887 to 100.0]	37.5 [18.5 to 61.4]	0.0009
Region (thigh/calf)	10.6 ± 0.99	0.084 ± 0.006	7.7% (3)	50.0% (3)	90.0 [77.0 to 96.0]	50.0 [18.8 to 81.2]	92.3 [79.7 to 97.4]	42.9 [15.8 to 75.0]	0.0370

^$^ based on Fisher’s exact test.

## Data Availability

Due to privacy and ethical review board rules, no data are made freely available but can be shared after direct author contact.

## Supplementary Materials

The following supporting information can be downloaded at: https://www.mdpi.com/article/10.3390/curroncol30030252/s1, Table S1: Overview of evaluated demographic and lesion parameters.
