# Supplementary material for: Evaluation of Classic and Quantitative Imaging Features in the Differentiation of Benign and Atypical Lipomatous Soft Tissue Tumors Using a Standardized Multiparametric MRI Protocol: A Prospective Single-Centre Study in 45 Patients
Source: Curr Oncol. 2023 Mar 13;30(3):3315–28. doi: 10.3390/curroncol30030252 (PMC10047222; doi:10.3390/curroncol30030252)
Supplement: Supplementary file 1 [file curroncol-30-00252-s001.zip › curroncol-2179295-supplementary.pdf]

Supplementary Table S1: Overview of evaluated demographic and lesion parameters

**Parameter**	**Levels**
DEMOGRAPHY	
Age	years
Sex	male/female
IMAGING	
Affected region	head, neck, shoulder, upper arm, lower arm, hand, chest, back, abdominal wall, genital area, thigh, knee, lower leg, foot
Fascial relationship	epifascial, subfascial, both
Diameters	longest, orthogonal longest and shortest axis
Volume	mm^3
Circularity	ratio of area and perimeter based on the longest and shortest tumour axis, according to Ramajunan's approximation for the ellipsis' perimeter
Sphericity	ratio of tumour surface area to the surface area of sphere with the same volume
Border definition	sharp, partially diffuse, diffuse
Border contour	smooth, lobulated
Altered septation morphology: architecture, density, thickening (compared to surrounding fat tissue)	absent/present
Maximum septation thickness	mm
Contrast enhancement pattern	none, band-like, patchy or 'dirty', nodular, whole lesion
T2 STIR pattern	none, band-like, patchy or 'dirty', nodular, whole lesion
STIR polar sparing	absent/present
STIR surrounding fluid	absent/present
Intralesional T2 STIR hyperintensity without surrounding fluid	absent/present
ADC pattern	none, band-like, patchy or 'dirty', nodular, whole lesion
Average ADC value	mm^2/s
